# Chronic Intermittent Hypoxia-Induced Aberrant Neural Activities in the Hippocampus of Male Rats Revealed by Long-Term *in vivo* Recording

**DOI:** 10.3389/fncel.2021.784045

**Published:** 2022-01-21

**Authors:** Linhao Xu, Qian Li, Ya Ke, Wing-Ho Yung

**Affiliations:** ^1^School of Biomedical Sciences, Faculty of Medicine, The Chinese University of Hong Kong, Hong Kong, Hong Kong SAR, China; ^2^Department of Cardiology, Affiliated Hangzhou First People's Hospital, Zhejiang University School of Medicine, Hangzhou, China; ^3^Gerald Choa Neuroscience Centre, Faculty of Medicine, The Chinese University of Hong Kong, Hong Kong, Hong Kong SAR, China

**Keywords:** chronic intermittent hypoxia, obstructive sleep apnea, *in vivo* recording, neurocognitive impairment, neural firing

## Abstract

Chronic intermittent hypoxia (CIH) occurs in obstructive sleep apnea (OSA), a common sleep-disordered breathing associated with malfunctions in multiple organs including the brain. How OSA-associated CIH impacts on brain activities and functions leading to neurocognitive impairment is virtually unknown. Here, by means of *in vivo* electrophysiological recordings via chronically implanted multi-electrode arrays in male rat model of OSA, we found that both putative pyramidal neurons and putative interneurons in the hippocampal CA1 subfield were hyper-excitable during the first week of CIH treatment and followed by progressive suppression of neural firing in the longer term. Partial recovery of the neuronal activities was found after normoxia treatment but only in putative pyramidal neurons. These findings correlated well to abnormalities in dendritic spine morphogenesis of these neurons. The results reveal that hippocampal neurons respond to CIH in a complex biphasic and bidirectional manner eventually leading to suppression of firing activities. Importantly, these changes are attributed to a larger extent to impaired functions of putative interneurons than putative pyramidal neurons. Our findings therefore revealed functional and structural damages in central neurons in OSA subjects.

## Introduction

Obstructive sleep apnea (OSA), a common sleep-disordered breathing, is associated with intermittent hypoxia resulting from upper airway obstruction of structural or neural causes (Mathieu et al., 2008). The most distinct features of OSA are episodes of oxyhemoglobin desaturations, which are terminated by brief microarousals that result in sleep fragmentation and alteration in sleep pattern (Deegan and McNicholas, 1995). The impact of OSA on neurocognitive performance has been well-documented, including impairment in attention, perception, memory, executive functions and also behavioral problems in children (Ali et al., 1996; Chervin et al., 1997; Gozal, 1998; Gozal et al., 2001; Row et al., 2002).

Many of the neurocognitive deficits found in OSA are consistent with malfunctions of the temporal lobe, including the hippocampal and para-hippocampal region, and other cortical areas such as the prefrontal cortex (Beebe and Gozal, 2002; Rosenzweig et al., 2015). In the past, investigators largely relied on neuroimaging techniques, especially magnetic resonance imaging (MRI), to investigate brain changes in OSA subjects. Significant changes in the gray matter and white matter are often found in the hippocampal area in both adult and children OSA subjects (Macey et al., 2002; Morrell et al., 2010; Torelli et al., 2011; Cha et al., 2017; Song et al., 2018; Owen et al., 2019). However, how intermittent hypoxia that occurs in OSA impacts on the neurons in the hippocampus or other brain areas are far from clear.

Animal models have been indispensible in advancing our understanding of the pathophysiology of OSA. For example, studies on rodents have shown that chronic intermittent hypoxia treatment, as a model of OSA, could impair spatial memory functions of the animals to different degrees (Goldbart et al., 2003a,b; Kheirandish et al., 2005a,b; Tartar et al., 2006; Ward et al., 2009). These results are consistent with studies showing that hippocampal long-term synaptic plasticity is impaired after chronic intermittent hypoxia treatment (Payne et al., 2004; Xie et al., 2010; Xu et al., 2015). However, the key question of how episodes of hypoxia affect the activity and therefore functions of hippocampal neurons in the intact brain is still unsolved.

In this study, we performed real-time and long-term recordings from neuronal ensemble in hippocampal CA1 region of rats at single cell and population levels during and after a chronic intermittent hypoxia paradigm. We found complex effects of intermittent hypoxia on the activities of principal neurons and interneurons in CA1. In addition, we correlated these results with the effects of chronic intermittent hypoxia on morphogenesis of dendritic spines of hippocampal neurons. Our findings help identify the cellular correlates of impaired hippocampal function imposed by chronic intermittent hypoxia that occurs in OSA and possibly other related pathological conditions.

## Materials and Methods

### Animals

A total of 29 six-week old male Sprague Dawley rats weighing 220–250 g were used in the experiments, including 14 control and 15 chronic intermittent hypoxia rats. The animals were housed under standard laboratory conditions, namely 12 h light/dark cycle at 22–24°C, with food and water provided *ad libitum*. The procedures of experimentation were approved by the Animal Experimentation and Ethics Committee of the Chinese University of Hong Kong.

### Implantation of Microwire Electrode Recording Array

Eleven rats (five controls and six IH) were used for implanting microwire electrode. Before electrode implantation, the rats were deeply anesthetized with pentobarbital sodium salt (Sigma-Aldrich, Darmstadt, Germany) at the dosage of 50 mg/kg by intraperitoneal injection. To record multi-unit neuronal activities and local field potentials *in vivo*, a multi-channel recording array consisting of 16 stainless steel Teflon-coated microwires of 50 μm diameter, arranged in 4 x 4 and measured ~1.1 x 1.1 mm^2^ (Plexon Inc, Dallas, TX), was implanted unilaterally targeting at hippocampal CA1 region according to standard stereotaxic atlas (center position: AP, −3.5 mm, ML, −2.0 mm, DV, 2.05 mm from dura). Four stainless steel screws were firmly attached to the skull for electrode anchoring, and an additional ground wire was connected to one of them as reference. The whole electrode array was secured with dental cement.

### Rat Model of Chronic Intermittent Hypoxia

After two weeks of recovery from the implantation surgery, a rat was put in a specially designed chamber (46 × 20 × 22 cm) and exposed to intermittent hypoxia environment under the control of an oxygen profiler (Oxycycler model A48XOV; Reming Bioinstruments, Redfield, NY). The hypoxia paradigm consisted of cycles of oxygen levels between 10 and 21% every 90s, i.e., 40 cycles/h, pioneered by Gozal's group which demonstrated its effectiveness decreasing pO_2_ values during the hypoxic cycles (Gozal et al., 2001). This or highly similar protocol has since been widely adopted (e.g., Goldbart et al., 2003a,b; Ma et al., 2008), including in our previous works (Xie et al., 2010; Xu et al., 2015). The hypoxia treatment was given during the daytime for 8 h, from 09:00 to 17:00 while the temperature, humidity and CO_2_ level inside the chamber were monitored by remote sensors. The temperature and humidity were maintained at 22–24°C and 40–50% respectively. Ambient CO_2_ in the chamber was periodically monitored and maintained at 0.03% by adjusting the overall chamber basal ventilation. The hypoxia treatment was given during the daytime for 8 h, from 09:00 to 17:00 for two weeks. After 2 weeks of IH treatment, the animals were returned to normoxia condition for 1 week.

### *In vivo* Electrophysiological Recording

Both the extracellular single-unit activities and local field potentials in the hippocampal CA1 subfield were recorded simultaneously by the 32-channel OmniPlex® system (Plexon Inc., Dallas, TX). Continuous spike signals were amplified (× 2500–3000), band-pass filtered (300 Hz to 5 k Hz, 4-pole Bessel) and sampled at 40 k Hz. For the control rats, data were recorded 5 min before IH. For the IH rats, the recorded data included 5 min before IH, the first of 5 min of IH and another 5 min after 8 h IH.

In order to avoid problems associated with responses to initiation of IH, all animals, including control rats and IH rats, were put into the CIH chamber for acclimatization with the environment two days before IH experiments. In addition, at the first and second day of IH treatment, several cycles of changing oxygen were given but with the door of the chamber opened, to minimize the behavorial and physiological responses to the flushing of gas and associated sounds. Real IH and recording started after this procedure.

### Spike Sorting and Long-Term Stability Analysis

Off-Line Spike Sorter (Version 3, Plexon Inc., Dallas, TX) was used for analysis of electrophysiological data by using a combination of automatic and manual sorting techniques (Li et al., 2012, 2017). A minimum waveform amplitude threshold of 3SDs higher than the noise amplitude was detected as a spike in each channel for analyzing the continuous spike trains. The first three principal components (PC) of all waveforms recorded from each channel were depicted in 3-dimensional (3D) space. Initially, individual clusters were separated by automatic clustering techniques (K-means clustering and valley seeking methods) based on the unit waveform (Li et al., 2012, 2017). Each cluster was then checked manually to ensure that the cluster boundaries were well-separated and the spike waveforms were consistent in each day. A similar waveform in each channel was considered as being generated from a single neuron only if it defined a discrete cluster in 2D/3D PC space. Each cluster was termed as a “single-unit” which was different from another. In addition, single-units had a characteristic waveform and exhibited a clearly recognizable refractory period (≥1 ms) in its inter-spike interval (ISI) histogram.

The stability of units throughout the experiment was confirmed by plotting the PC1 and PC2 vs. the time stamp for each waveform. Furthermore, three different statistics were used to objectively quantify the overall separation between identified clusters in a certain channel. These evaluation indexes included the classic F, the J3 and the Davies-Bouldin (DB) validity index (Nicolelis et al., 2003). F is a parametric statistics of multivariate analysis of variance (MANOVA), J3 is a measure of the ratio of between-cluster to within-cluster scatter, and DB is a measure of the ratio of the sum of within-cluster scatter to between cluster separations. Channels with high J3 and F statistics and low DB values indicate the presence of well-separated clusters. Although none of these measures alone provided the optimal criterion for separating clusters, using them in conjunction improves the quality of unit isolation. Based on the offline sorting result, multi-unit spikes generated by both principal, pyramidal and interneurons could be recognized. Pyramidal neurons and interneurons were identified based on their waveforms and temporal firing characteristics (Csicsvari et al., 1999; Hussaini et al., 2011). In order to validity of the long-term study, the “single unit” which consistently appeared in each detection without waveform change was included for analysis while those which were separated in the first time of detection but disappeared subsequent recording days were excluded.

### Golgi–Cox Staining

Golgi–Cox staining was performed using the FD Rapid GolgiStain kit (FD NeuroTechnologies) according to the instruction of the manufacturer. Neurons from the CA1 region of the hippocampus were observed under a light microscope (Zeiss Microscope Axiophot 2, USA) by an investigator blind to the treatment. 18 rats (nine control and 9 IH, three rats for each time point, including Day 1, Day 14 and Day 21) were sacrificed to assess dendritic change. Pyramidal neurons and interneurons were identified according to the location of the cell body and the morphology of dendrites (Klausberger and Somogyi, 2008). The spines, classified as stubby, mushroom or thin subtypes based on well-defined criteria (Harris et al., 1992). Three rats were used in each group, and 15 putative pyramidal neurons (54 dendrites) and 12 putative interneurons (45 dendrites) of each group were selected.

### Statistical Analysis

Results are displayed as box plots, and in each box, the central mark indicates the median, and the bottom and top edges of the box indicate the 25th and 75th percentiles, respectively, while mean ± SEM are used in **Figures 2A,B**, **3A,B**, **4**. Paired Student's *t*-test was performed on the data from the same animal for two different time points except firing rates of pyramidal neurons which were analyzed by Wilcoxon's paired signed rank test. Unpaired Student's *t*-tests were used to compare between two different groups. Repeated-measures ANOVA and the Newman–Keuls *post-hoc* test were applied to compare values from the same group or more than two time points expect analyzing firing rate of pyramidal neurons which was performed by Repeated-measures ANOVA (Friedman test) and Dunn's multiple comparisons *post-hoc* test. One-way ANOVA and the Newman–Keuls *post-hoc* test were applied to compare values from the multiple groups.

## Results

### Characterization of Neural Activities in CA1 During Prolonged *in vivo* Recordings

**Figure 1:**
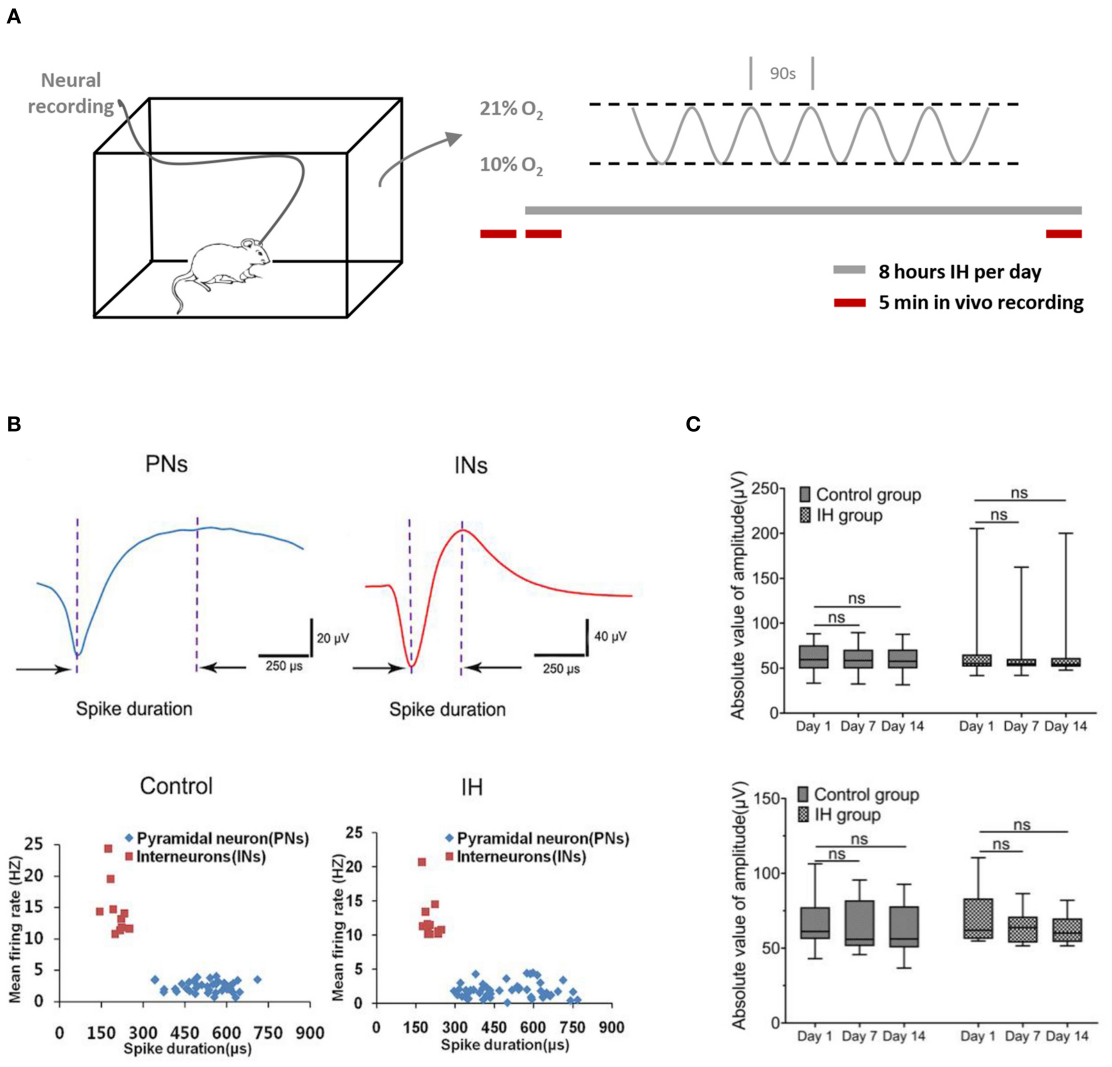
Characterization of neural activities in hippocampl CA1 sub-field during prolonged *in vivo* recordings. **(A)** (Left) Ventilated cages that mimic typical housing conditions were used to expose the experimental rats to intermittent hypoxia paradigms while recording the neural activities from the hippocampus. (Right) The intermittent hypoxia pattern consists of cyclic oscillations of O2 between 21 and 10% within 90 s during the 8-h daylight. Five-min recording sessions was conducted which were immediately before, at the beginning and also at the end of the daily 8-hr hypoxia treatment during the entire 2-weeks study. **(B)** Recorded neurons were classified into putative pyramidal neurons (PNs) and putative interneurons (INs) based on their electrophysiological properties. (Top) Two typical single-units representing PN (blue) and IN (red) respectively. (Bottom) Scatter plots depicting the distribution of mean firing rate vs. spike width of all PNs and INs recorded from the CA1 region of hippocampus in control and intermittent hypoxia (IH) group. Compared with putative pyramidal neurons, putative interneurons exhibited spikes with shorter spike width but higher firing rates. **(C)** Repeated measure ANOVA revealed that the amplitudes of putative pyramidal neurons (top) and putative interneurons (bottom) during the 2 weeks showed no significant changes.

To mimic OSA-associated intermittent hypoxia, rats were placed in specially designed chambers and exposed to intermittent hypoxia paradigm consisting of cycling oxygen levels between 10 and 21% in 90s for 8 h (Figure 1A). Neural activities from hippocampal CA1 regions were recorded at specified time via prior implantation of multi-electrode arrays (Figure 1B; Supplementary Figure 1A). Extracellular action potentials originating from individual units were identified and sorted by their characteristic waveforms, and checked for their long-term stability (Supplementary Figure 2). Based on the duration of the spikes and their frequencies, two populations of neurons were found with properties that are consistent with the principal, pyramidal neurons (lower frequency of <5 spikes/s; spikes with broad widths with peak-to-trough width > 0.3 ms) and interneurons (higher frequency of > 5 spikes/s; spikes with shorter widths with peak-to-trough width < 0.3 ms) reported previously (Csicsvari et al., 1999; Hussaini et al., 2011). As shown in Figure 1B, putative pyramidal neurons (five rats, 41 neurons in control group; six rats, 50 neurons in IH group) have relatively long spike duration of more than 0.3 ms (0.53 ± 0.01 ms in control group, 0.51 ± 0.02 ms in IH group,) and a mean firing frequency <5 Hz (2.43 ± 0.13 spikes/s in control group, 1.86 ± 0.16 spikes/s in IH group; 50 cells). On the other hand, putative interneurons (5 rats, 10 neurons in control group; six rats, 12 neurons in IH group) have shorter spike duration of <0.3 ms (0.20 ± 0.01 ms in control group; 0.21 ± 0.02 ms in IH group) and a mean firing rate of more than 10 Hz (14.60 ± 1.35 spikes/s in control group; 12.15 ± 0.87 spikes/s in IH group).

To ensure the validity of our approach in elucidating the long-term effect of intermittent hypoxia on neuronal activities, we performed stringent tests for inclusion of units for analysis (see Methods; Supplementary Figures 1, 2). Examples of stable single-unit recordings during the 2 weeks are shown in Supplementary Figure 2. In this report, data presented were obtained from 41(80.4%) and 50 (85.1%) putative pyramidal neurons, and also 10 (19.6%) and 12 (14.9%) putative interneurons, in the control and hypoxia group respectively that met our criteria of recording stability (Supplementary Figure 1B). As summarized in Figure 1C, the stability was also reflected in the stable mean amplitudes of the extracellular spikes in both control and hypoxia groups in day 1, day 7 and day 14, for both putative pyramidal neurons [control group: *F*_(1.41, 56.46)_ = 1.75, *n* = 41, from five rats, *P* = 0.19; IH group: *F*_(1.47, 72.25)_ = 0.49, *n* =50, from six rats, *P* = 0.56; top] and putative interneurons [control group: *F*_(1, 69, 15.17)_ = 1.40, *n* = 10, from five rats, *P* = 0.27; IH group: *F*(1,35, 14.81) = 4.28, *n* =12, from six rats, *P* = 0.05; bottom]. Therefore, we concluded that we could reliably track the activities of the recorded neurons throughout the periods studied.

### Acute and Long-Term Effects of Intermittent Hypoxia on the Firing of CA1 Putative Pyramidal Neurons

We conducted 5-min recording sessions immediately before, at the beginning and also at the end of the daily 8-h hypoxia treatment during the entire 2-weeks study. This design enabled us to determine the acute as well as prolonged effects of intermittent hypoxia on neuronal excitability and population activities in the hippocampus. In addition, we could assess the sustained effects of intermittent hypoxia after the subjects had been re-exposed to normal oxygen levels, and also the accumulated effects of intermittent hypoxia over days and weeks. Control animals received the same handling procedures except the hypoxic treatments. During the recording in the daytime that corresponded to the inactive phase of the daily cycle, the animals were mainly resting and largely immobile.

**Figure 2:**
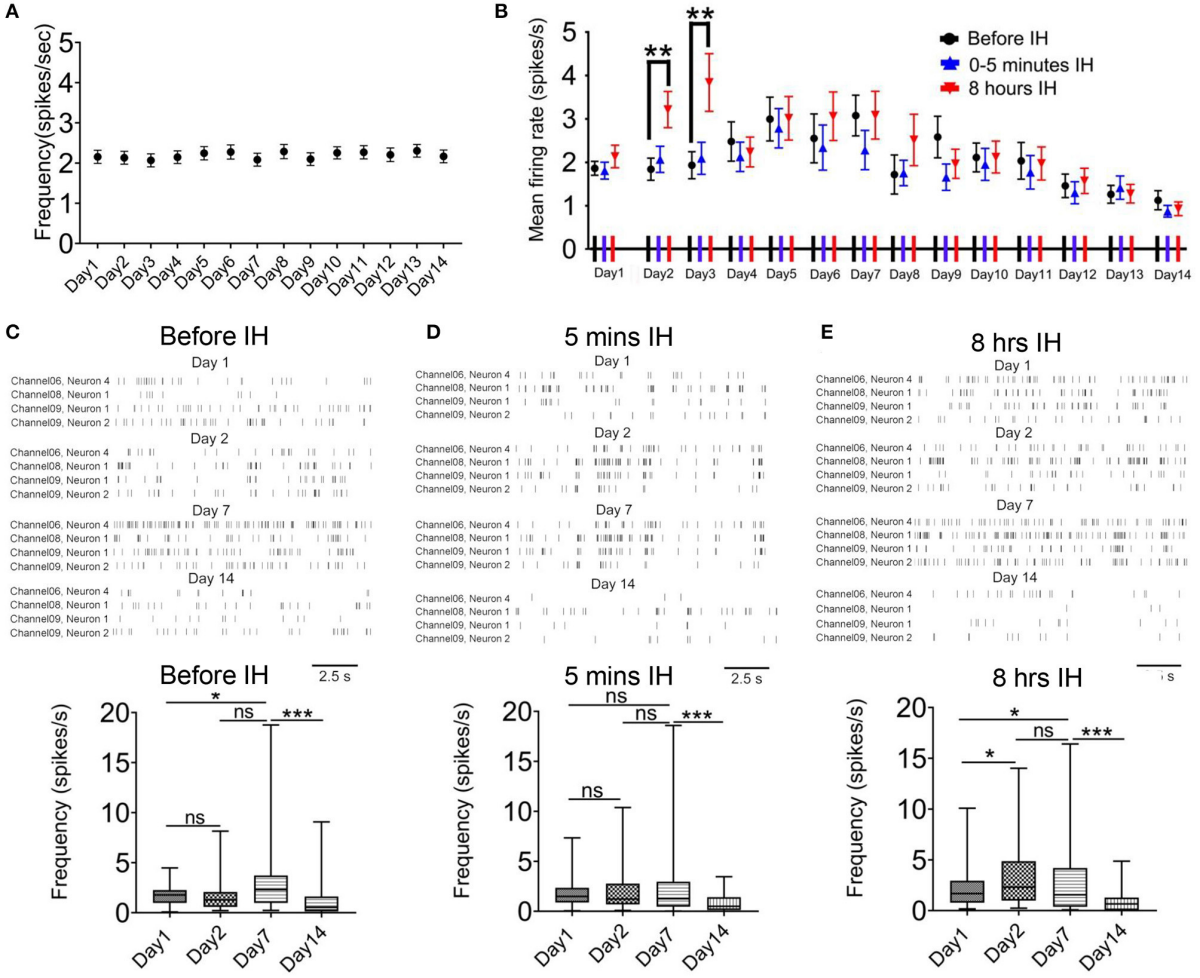
Intermittent hypoxia (IH) exerts complex acute and long-term effects on the firing activities of CA1 putative pyramidal neurons. **(A)** Repeated measured ANOVA revealed that neuronal firing was not significantly affected in control group which was recorded at about 9:00–9:05 a.m. **(B)** Summary of the mean firing rates of putative pyramidal neurons recorded in the 3 recording sessions in each day over the 2-week period. Putative pyramidal neurons in CA1 region tended to increase their firing activities during the first week of IH treatment, particularly apparent at the late hours (at 8 h) but not early hours (at 5 min and 3 h) of daily IH paradigm. Repeated measured ANOVA revealed that neuronal firing was significantly affected in the 2nd and 3rd day. The hyper-excitability, however, was followed by gradual suppression of firing in the second week. **(C)** Typical raster plots of the CA1 putative pyramidal neurons recorded before IH on day 1, 2, 7 and 14 (up panel). Statistical results are shown on the bottom. **(D)** Typical raster plots of the CA1 putative pyramidal neurons recorded in the first 5 IH min of IH treatment on day 1, 2, 7 and 14 are shown on the up and the mean data are summarized on the bottom. **(E)** Typical raster plots of the CA1 putative pyramidal neurons recorded in the last 5 IH min of IH treatment are shown on the up and the mean data are summarized on the bottom. *P < 0.05, ***P < 0.001.

We did not find any changes in the firing rate of the control animals in the morning hours (08:55–09:00) during 2-week recording (Friedman statistic = 22.08, *n* = 41, from five rats, *P* = 0.05; Figure 2A). In the case of intermittent hypoxia-treated group, Figure 2B summarizes the mean firing rates of putative pyramidal neurons in the 3 recording sessions in each day over the 2-week period. We observed a complex profile of neuronal firing, which was dependent on the time of recording (namely before, at the start, or at the end of hypoxia treatment) and also the days of treatment. First, we found that within the daily hypoxia episode, the firing of the neurons in the first 5 min of intermittent hypoxia was not affected. In contrast, in the 2nd and 3rd day, 8 h of prolonged intermittent hypoxia treatment significantly increased the firing rates of these neurons (Friedman statistic = 23.08, *n* = 50, from six rats in 2nd day, *P* < 0.001; Friedman statistic = 16.59, n = 50, from six rats in 3rd day, *P* < 0.001), although no significant effect was found in the other days. This increased firing under long hours of intermittent hypoxia did not persist overnight, and the firing rates returned to baseline values the next day (Figure 2B), indicating that these were transient rather than sustained effects.

Regarding the prolonged, accumulative effects of daily intermittent hypoxia on the baseline firing of the neurons, a bi-phasic and bi-directional response was observed. Thus, we observed a general increase in firing of the neurons in the first week, followed by a gradual and finally significant decrease in firing activities (Figure 2B). Typical raster plots of the CA1 putative pyramidal neurons recorded before, during the first 5 min and the last 5 min of intermittent hypoxia on day 1, 2, 7 and 14 are shown in Figures 2C–E. First, it was found that the basal mean firing, that is, recordings made before the IH, was significantly increased at day 7 when compared with day 1 and then the firing rate was decreased at day 14 (Figure 2C). The basal firing rate before the intermittent hypoxia session on day 14 was only 60.5 % of that of day 1 (1.86 ± 0.16 spikes/s in day 1, 50 cells; 1.13 ± 0.22 spikes/s in day 14, 50 cells; *P* < 0.01). The increase in firing reached statistical significance at day 7 when compared with day 1 for recordings made before and also toward the end of the intermittent hypoxia. Second, the mean firing was significantly altered in the first 5 min IH on day 1, 2, 7 (Figure 1D). Finally, a clearly elevated firing could be found on day 2 and 7, but this effect was not apparent in day 14, in which the firing was significantly suppressed (Figure 2E).

### Effects of Intermittent Hypoxia on the Firing of Putative Interneurons in CA1

**Figure 3:**
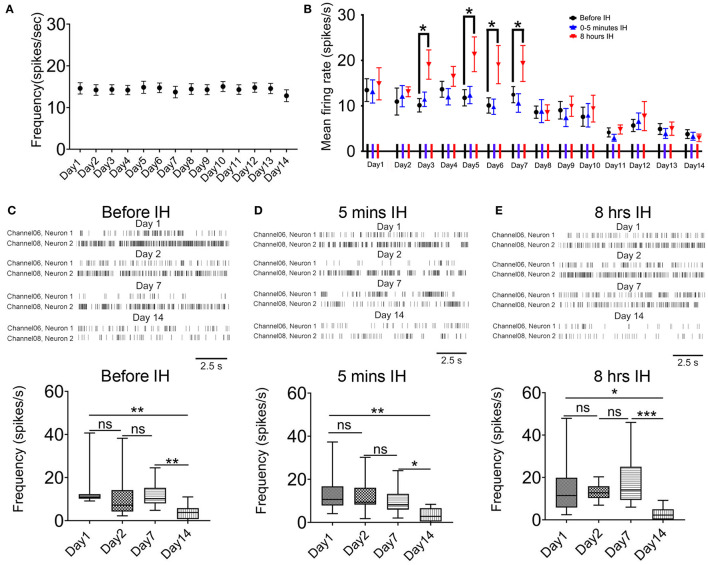
Acute and long-term effects of intermittent hypoxia (IH) on the firing of CA1 putative interneurons. **(A)** Repeated measured ANOVA revealed that neuronal firing was not significantly affected in control group. **(B)** Summary of the mean firing rates of putative interneurons recorded in the three recording sessions in each day over the 2-week period. Apart from the significantly elevated firing of the putative interneurons after 8 h of IH treatment in some days of the first week, the major effect of the chronic intermittent hypoxia was a strong suppression of firing found in the second week of IH treatment. **(C)** The up panel shows typical raster plots of the putative interneurons recorded before IH on day 1, 2, 7 and 14. Statistical results are shown on the bottom. **(D)** Typical raster plots of the putative interneurons recorded in the first 5 IH min of IH treatment on day 1, 2, 7 and 14 are shown on the up and the mean data are summarized on the bottom. **(E)** Typical raster plots of the putative interneurons recorded in the last 5 IH mins of IH treatment on day 1, 2, 7 and 14 are shown on the up and the mean data are summarized on the bottom. *P < 0.05, ** *P* < 0.01, ***P < 0.001.

We also analyzed the effects of chronic intermittent hypoxia on the interneurons in the same area. First, in the control animals, the mean firing rate in putative interneurons did not change significantly during the 2-weeks ([*F*_(1.40, 12.60)_ = 0.38, *n* = 41, *P* = 0.62]; Figure 3A). Second, we observed a similar but not identical response pattern of these neurons with that of putative pyramidal neurons. Figure 3B plots the profile of the responses of the putative interneurons to intermittent hypoxia spanning the 2 weeks. Similar to the putative pyramidal neurons, no acute changes in the firing rates of the putative interneurons were found in the first 5 min of intermittent hypoxia, regardless of the number of days of treatment. We also found that, almost throughout the first week, continuous intermittent hypoxia for 8 h increased the firing rate of putative interneurons, with respect to the beginning of the intermittent hypoxia episode of the same day (Figure 2B). For example, repeated measured ANOVA showed that neuronal firing was significantly affected on day 3 [*F*_(1.15, 12.60)_ = 4.70, *n* = 12, from six rats, *P* < 0.05], day 5 [*F*_(1.10, 12.13)_ = 6.13, *n* = 12, from six rats, *P* < 0.05] and day 6 [*F*_(1.14, 12.50)_ = 7.59, *n* = 12, from six rats, *P* < 0.05].

Regarding the accumulated effects of daily intermittent hypoxia, different from the biphasic response of the putative pyramidal neurons, no upregulation of basal firing was observed but only a significant decrease in firing was found in the second week. Representative raster plots of putative interneurons at the different sampling time and days are shown in Figures 3C–E. First, the basal firing, that is, recordings made before the IH, was significantly decreased at day 14 when compared with day 1 (Figure 3C). On day 14, only 28.1% of basal firing remained, which represents a much bigger suppression in neural activities compared with that of the putative pyramidal neurons [*P* < 0.01; 13.46 ± 2.50 spikes/s in day 1, 12 cells; 3.78 ± 0.97 spikes/s in day 14, 12 cells; Figure 2C). Second, there was no significant difference in firing rates among day 1, 2 and 7 in the first 5 min IH but a significant reduction was found in the same period in day 14 (Figure 3D). Finally, when recordings were made 8 h after IH and compared among different days, only a significant reduction in firing rate was detected on day 14 by Newman-Keuls's test.

Furthermore, neuronal discharges between control and CIH rats were also compared across exposure time), which confirmed significant decrease in neuronal firing of PNs (Supplementary Figure 3A) and INs (Supplementary Figure 3B) toward the second week.

### Real-Time Response of Neuronal Firing With Hypoxic Cycle

Under the condition of sleep apnea, there are cyclic changes of the oxygenation level in the circulation, and therefore oxygen supply to the brain. One question of interest is whether the level of oxygenation has a real-time impact on the activities of brain neurons. Although the level of oxygen in the vicinity of the neurons under recording cannot be measured, studying the temporal relationship between the ambient air oxygen level and the instantaneous firing rate of the neurons may provide insight into this question.

**Figure 4:**
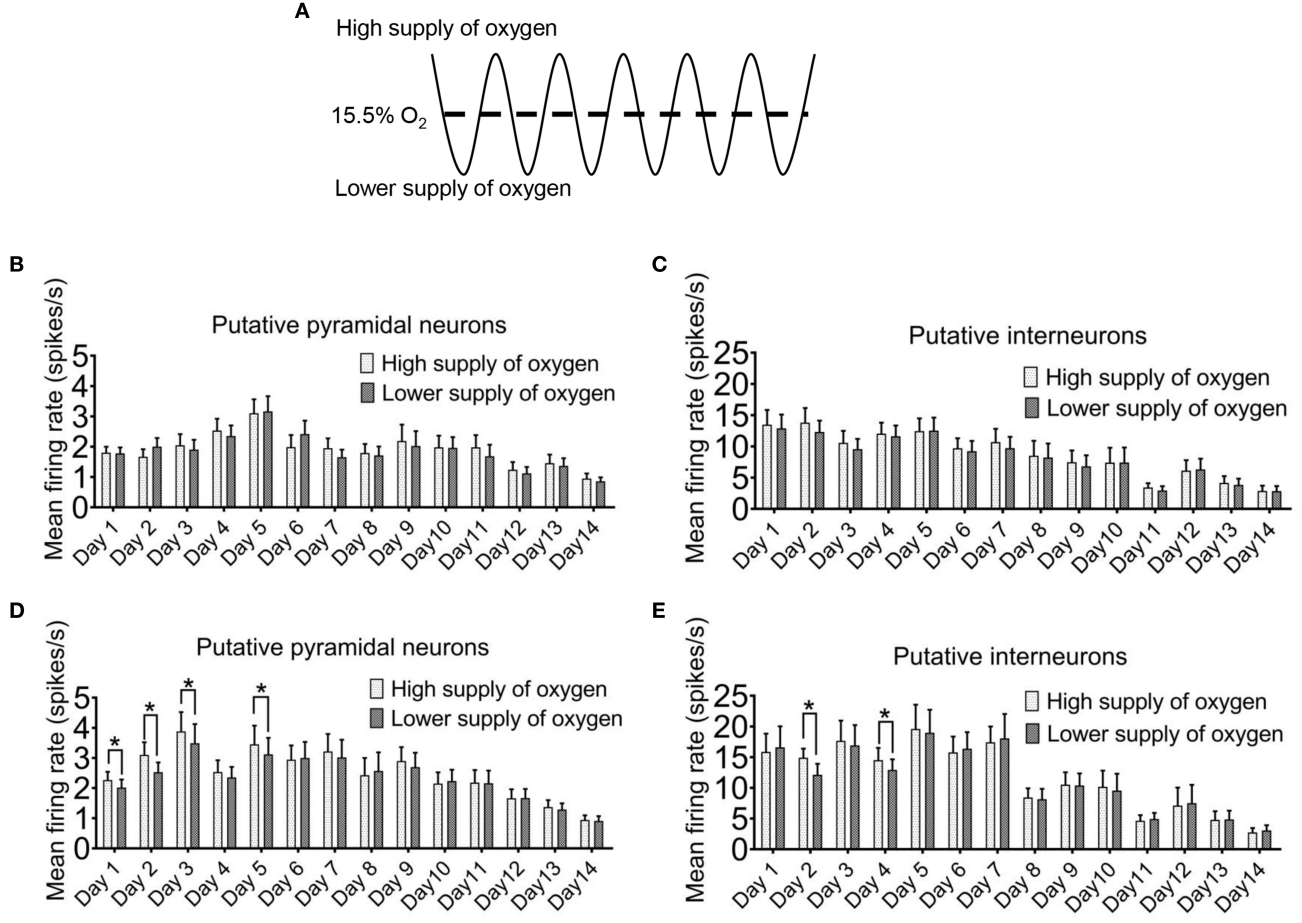
Instantaneous firing of hippocampal neurons can be affected by the ambient oxygen level in the hypoxic cycle. **(A)** The time period during which the ambient O_2_ level was above the mean (15.5%) was regarded as high supply of O_2_, while the time period during which the O_2_ was below the mean was designated as lower supply of O_2._ In the first 5 min of intermittent hypoxia (IH), the level of oxygen supply had no clear effect on the firing of both the putative pyramidal neurons **(B)** and the putative interneurons **(C)**. However, in the last 5 min of 8 h IH treatment, both putative pyramidal neurons **(D)** and putative interneurons **(E)** tended to fire more during the period of higher oxygen supply. The data were from 50 putative pyramidal neurons and 12 putative interneurons (six rats). Wilcoxon's paired signed rank test for pyramidal neurons; Paired Student's *t*-test for interneurons; *P < 0.05.

For most of the time, we did not find a clear temporal relationship between the firing rates of the neurons with the ambient oxygen level. When we compared the sampled mean firing rates of the recorded neurons when the oxygen level was above mean level (15.5%) with those below the mean level (Figure 4A), no differences were detected after 5 min of intermittent hypoxia (Figures 4B,C). The only exception was found at the times when the overall firing rates were elevated after 8 h of intermittent hypoxia in the first week (Figures 4D,E). Under this condition, in both pyramidal neurons and interneurons, the mean firing rates were lower when the ambient oxygen level was below the mean, that is, when there was a relatively low supply of oxygen. The average firing rate of all pyramidal neurons recorded in the phase of relatively high oxygen level (2.27 ± 0.27 spikes/s in day 1, *n* = 50 cells; 3.10 ± 0.42 spikes/s in day 2, *n* = 50 cells; 3.88 ± 0.64 spikes/s in day 3, *n* = 50 cells; 3.45 ± 0.62 spikes/s in day 5, *n* = 50 cells) was significantly higher than that in the phase of lower oxygen level (2.02 ± 0.26 spikes/s in day 1, *n* = 50 cells; 2.53 ± 0.32 spikes/s in day 2, *n* = 50 cells; 3.49 ± 0.63 spikes/s in day 3, *n* = 50 cells; 3.12 ± 0.54 spikes/s in day 5, *n* = 50 cells; *P* < 0.05). Although real-time pO_2_ or SaO_2_ values were not monitored in the freely moving rats, such cyclic changes in neuronal activities with an interval of 90s could be very obvious in some neurons (Supplementary Figure 4), consistent with the effect of the cyclic IH.

### Long-Lasting Efforts of Intermittent Hypoxia on Putative Interneurons

One important consideration of the adverse effects of chronic intermittent hypoxia on neural activity is whether they are long lasting or readily recoverable. Thus, in our study, after two weeks of intermittent hypoxia, we allowed some animals to breathe in normoxia condition for 1 week, and tracked the progress of recovery on neuronal firing in the hippocampus at 8:55–9:00 a.m.

**Figure 5:**
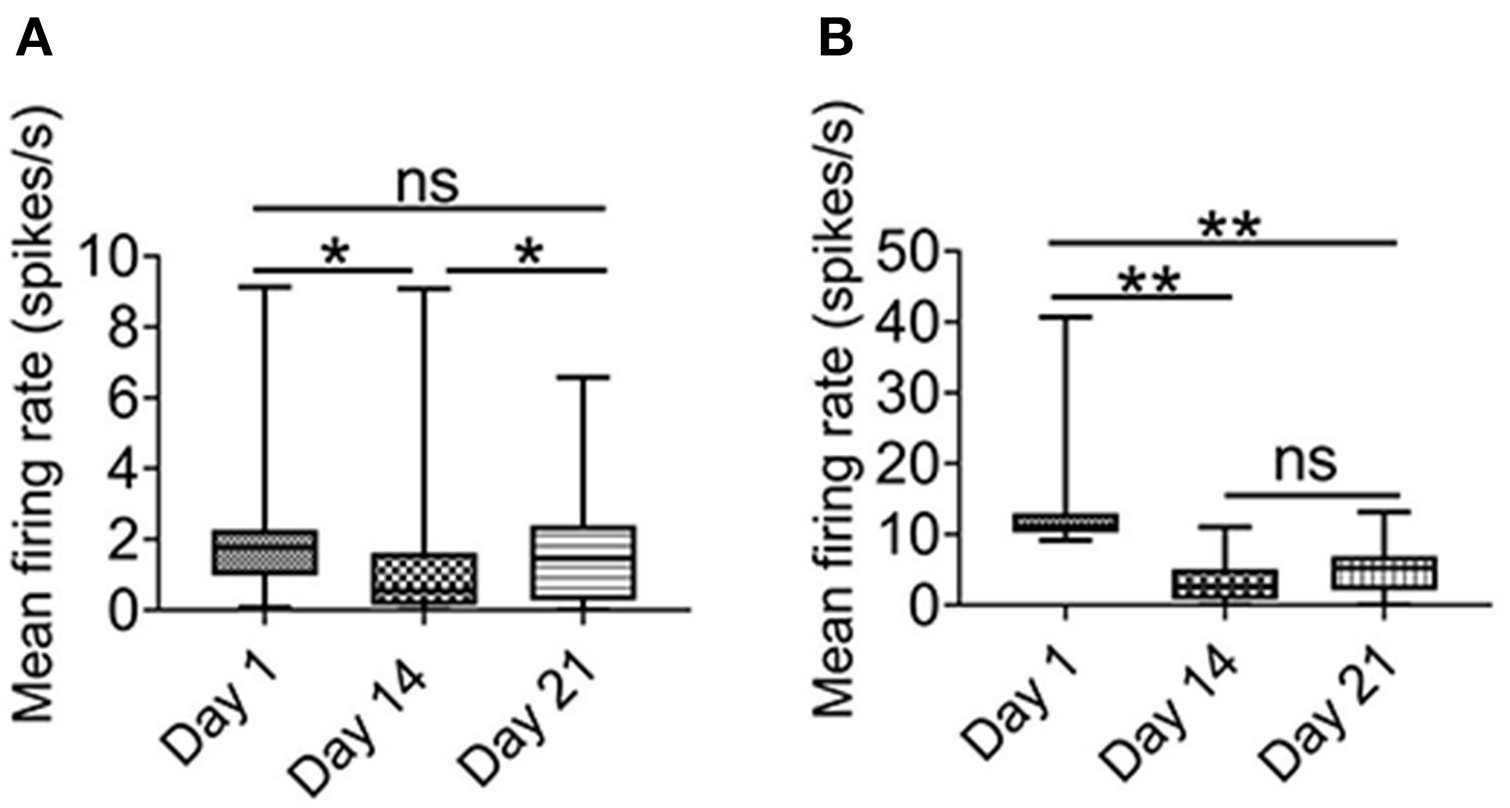
Effect of intermittent hypoxia on CA1 putative interneurons firing is long-lasting. **(A)** Repeated measured ANOVA showed that firing activities in putative pyramidal neurons was altered on day 1 (before IH treatment), day 14 (before IH treatment) and day 21 (Friedman statistic = 20.73, *n* =50, from six rats, *P* < 0.001). Post Dunn's multiple comparisons showed that significant recovery in the firing activities in putative pyramidal neurons after 1 week of normoxia. **(B)** Repeated measured ANOVA showed that firing rate was different among day 1 (before IH treatment), day 14 (before IH treatment) and day 21 [*F*_(1.25, 13.74)_ = 11. 98, *n* = 12, from six rats, *P* < 0.01], furthermore, *post-hoc* Newman-Keuls's test showed that no recovery of firing rate of putative interneurons was found 1 week after normoxia treatment. *P < 0.05, **P < 0.01.

As shown in Figure 5A, after 1 week of recovery, the firing rate of putative pyramidal neurons was largely recovered. In contrast, there was no significant recovery of the firing rate of the putative interneurons (Figure 5B). These results suggested that not only the putative interneurons were more sensitive to chronic intermittent hypoxia, but their activities could not be restored, at least one week later.

### Correlation to Spine Morphogenesis

**Figure 6:**
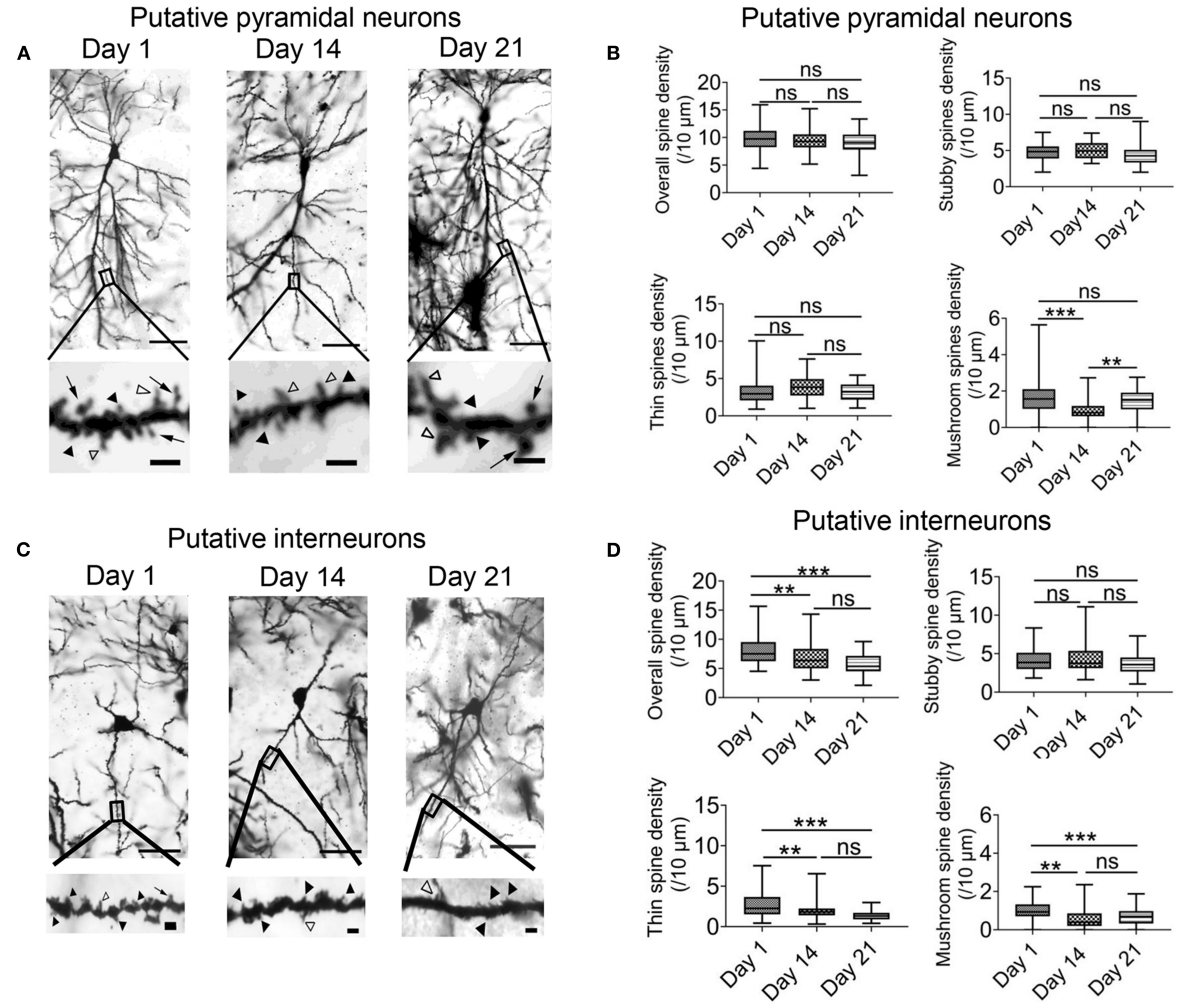
Chronic intermittent hypoxia (IH) reduces mature spine formation of CA1 pyramidal neurons and interneurons. **(A)** Typical morphologies of individual pyramidal neurons in CA1 region revealed by Golgi staining on day 1, 14 and 21. Scale bar: 50 μm. The higher magnification pictures shown in the lower panels allowed quantification of spine density and classification of individual spines. Black arrowheads indicate stubby spines; white arrowheads indicate thin spines; arrows indicate mushroom spines Scale bar: 4 μm. **(B)** Quantification analysis from 54 dendrites revealed no significant difference between day 1, 14 and 21 in terms of overall spine density (top left), stubby spine density (top right) and thin spine density (bottom left). However, the density of the mushroom spine was significantly altered (bottom right). Meanwhile, *post-hoc* Newman-Keuls's test revealed mushroom spine density was decreased significantly in 2 weeks IH treatment, which could recover on day 21 (right panel). ***P < 0.001. **(C)** Typical morphologies of individual interneurons in CA1 region revealed by Golgi staining on day 1, 14 and 21. Scale bar: 50 μm. Higher magnification pictures are shown in the lower panels. Scale bar: 4 μm. **(D)** Quantification of data from 45 dendrites showed that there was significantly difference in the overall spine density (top left), thin spine density (bottom left) and mushroom spine (bottom right) except stubby spine density (top right). *Post-hoc* Newman-Keuls's test revealed clear reduction of overall spine density after 2 weeks of IH treatment, compared with day 1, as well as the densities of thin spine and mushroom spine. These changes were not recoverable even after 1 week of normoxia treatment, **P < 0.01, ***P < 0.001.

The previous results indicated that intermittent hypoxia could exert both transient and persistent effects on the functions of hippocampal CA1 neurons. We therefore also examined the morphology of dendritic spines in the pyramidal neurons and interneurons by the end of the 2-week hypoxia treatment as well as in the recovery phase. Based on Golgi staining, pyramidal neurons and interneurons could be readily distinguished according to their cellular morphology as well as somatic locations (Supplementary Figure 5) (Klausberger and Somogyi, 2008). As demonstrated in Figures 6A,B, there was no significant change in the overall spine density of pyramidal neurons between day 1 group (9.72 ± 0.33 spines/10 μm, 54 dendrites) and 2 weeks intermittent hypoxia group (9.43 ± 0.28 spines/10 μm, 54 dendrites). Further analysis, however, revealed that the mushroom spine density was significantly reduced in 2 weeks intermittent hypoxia group (0.97 ± 0.09 spines/10 μm, 54 dendrites) when compared with day 1 group (1.71 ± 0.14 spines/10 μm, 54 dendrites) while no significant difference was found for the stubby and thin spines (Figure 6B). On the other hand, consistent with the functional data, we found that interneurons were vulnerable, as the overall density of the spines (6.83 ± 0.34 spines/10 μm, 45 dendrites), as well as mushroom spines (0.61 ± 0.09 spines/10 μm, *n* = 45) and thin spines (2.00 ± 0.16 spines/10 μm, 45 dendrites) were all decreased after 2 weeks of intermittent hypoxia treatment compared with that of day 1 group (overall spines density: 8.01 ± 0.36 spines/10 μm; mushroom spines density: 0.99 ± 0.08 spines/10 μm; thin spines density: 2.72 ± 0.26 spines/10 μm, 45 dendrites, Figures 6C,D).

Finally, the change in the density of the spines in pyramidal neurons was recoverable after one week of normoxia treatment (Figure 6B). In contrast, in the interneurons, the decrease in the overall spine density, thin spines and mushroom spines were not restored despite one week of normoxia period (Figures 6C,D). These results further support that alterations of neuronal activities in the hippocampus are more related to impaired function and structure of interneurons.

## Discussion

The nervous system has a high energy demand and is therefore particularly sensitive to oxygen supply (Jiang and Haddad, 1994; Ances et al., 2008). Although there exists in the literature a large volume of data suggesting structural and functional deficits in the hippocampus of OSA subjects and animal models (Macey et al., 2002; Morrell et al., 2010; Torelli et al., 2011; Cha et al., 2017; Song et al., 2018; Owen et al., 2019), the impact of chronic intermittent hypoxia on the excitability of hippocampal neurons is virtually an untackled question. The present attempt is the first to systematically investigate both the acute and accumulated effects of intermittent hypoxia on the neuronal firing rate of hippocampal neurons in the intact brain. In terms of individual neurons in CA1 sub-field, we found that there are intricate and multiple phases of response to intermittent hypoxia treatment. CA1 putative pyramidal neurons tended to increase their firing activities during short-term (i.e., within 1 week) exposure to daily intermittent hypoxia. During this period, an episode of intermittent hypoxia for hours also increased the sensitivity of both putative pyramidal neurons and putative interneurons to ambient oxygen levels, resulting in exaggerated and probably aberrant firing activities. These phenomena were then followed by a progressive decline in firing in the second week finally leading to strong suppression of neural activities. Recovery of the neuronal activities was evident after 1-week recovery, only for putative pyramidal neurons but not putative interneurons.

Given the known heterogeneity in response to hypoxia and the different time courses of distinct adaptive or regulatory processes (Richter et al., 1991; Bickler and Donohoe, 2002; Pena and Ramirez, 2005), our finding of a complex response to chronic intermittent hypoxia is not entirely surprising. Although the exact causes of the biphasic, bi-directional responses are unclear and are beyond the scope of the present study, one may speculate that the increase in activity during the short-term intermittent hypoxia treatment could be related to altered neurotransmission, e.g., as a consequence of enhanced glutamate release (Hansen, 1985; Vangeison and Rempe, 2009) or other factors such as increased release of brain-derived neurotrophic factor (Vermehren-Schmaedick et al., 2012), consistent with increased expression of c-fos (Ma et al., 2008; Sharpe et al., 2013). On the other hand, the decrease in excitability after a long-term exposure to daily intermittent hypoxia may reflect the cellular damages caused by elevated level of oxidative stress (Kim et al., 2013) and ER stress (Xu et al., 2015), or represents an adaptive response important for the survival of the neurons, at the cost of reduced neuronal function (Gavello et al., 2012). Meanwhile, more prolonged IH could trigger other adaptive responses such as down-regulation of Na-channels (Gu and Haddad, 2001), which could confer some advantages in preventing excessive energy expenditure. The impaired activities could also be contributed by changes in the density of mature spines after 2 weeks of intermittent hypoxia treatment, which were evident in both pyramidal neurons and interneurons. Despite that a causal relationship between impaired activities and density of mature spines was not investigated in the present study, it had been demonstrated that NMDA and AMPA receptor existed in mature spines which could affect neuronal activity (Duman and Li, 2012; Wang et al., 2016).

It should be noted that hemodynamic compensatory changes may influence IH-mediated neural activities alteration. For example, higher respiration rhythm and instability (Chang et al., 2013) and a significant shift of the heart rate variability power spectrum, with a predominance of the sympathetic modulation (Iturriaga et al., 2010) were observed in OSA patients. In agreement, sustained sympathoexcitation and elevated arterial pressure present in OSA patients or after exposure to IH treatment had been found (Weiss et al., 2015). However, exactly how hemodynamic changes affect the neuronal firing of the hippocampal neurons would require further investigation.

Sleep fragmentation is an important factor in behavioral and functional defects in OSA patients. But one feature of the OSA model adopted in the present study is its dissociation from the impact of sleep fragmentation, as the hypoxia was induced not by respiratory blockade but by mimicking the resulting hypoxic cycles (Gozal et al., 2001). Therefore, in this study, we showed that in the absence of clear or significant sleep fragmentation, cyclic hypoxic episodes could affect hippocampal neuronal structures and functions.

Intriguingly, we found that there were no obvious changes in the activities of CA1 neurons in the first few minutes of intermittent hypoxia. This is in sharp contrast to previous *in vitro* studies which have shown that hippocampal neurons stop to generate action potentials in response to even brief period of hypoxia because of membrane depolarization or hyperpolarization affecting ion channel activities (Hansen et al., 1982; Hansen, 1985). Whether such tolerance is due to the involvement of anaerobic metabolism *in vivo* (Milton and Dawson-Scully, 2013) or is a reflection of the difference between intermittent and continuous hypoxia is not known.

Unlike the pyramidal neurons, the effect of intermittent hypoxia on interneurons has not been addressed before and thus their roles in mediating alteration of neuronal circuits in hypoxia are not known. An important finding of the present study is that, in the hippocampus, putative interneurons are more vulnerable to damage than putative pyramidal neurons in response to accumulative intermittent hypoxia. Not only that putative interneurons exhibit a much stronger suppression in neuronal firing, their activities also could not recover when the animals were allowed to breathe under normoxia for up to one week. Together, these observations implicate that dysfunctions of interneurons might contribute to a much larger extent than pyramidal neurons on neuronal circuit malfunctions under CIH.

There are several technical issues in this study that should be pointed out. Although control rats were also put into a chamber which was identical to the hypoxic chamber, no flushing of O_2_/NO_2_ was given to the control animals, which breathed normal O_2_. It is possible that flushing of gases may have a mild effect on neuronal activities of the IH animals. For the *in vivo* recording, Teflon-coated microelectrode arrays rather than tetrodes were used to track the activity of neurons. Although this may compromise the accuracy in the identification of single units, non-tetrode microarrays had also been used successfully for chronic recordings by others (Tseng et al., 2011) as well as in our previous study (Li et al., 2017), fulfilling multiple criteria. Furthermore, in the present study, the animals were singly housed in the present study, which may have imposed some stress on them and influencing activities of hippocampal neurons (Irvine and Abraham, 2005). Also, only male rats were used in this study since it was reported that the prevalence of OSA was higher in men when compared with women (Lurie, 2011; Kang et al., 2014). Although the reason remains obscure, hormonal factor has been considered to contribute to gender difference in OSA prevalence. Further investigation is needed to determine if there is any difference in the response of the hippocampus to chronic intermittent hypoxia between male and female rats. Finally, although memory deficits would be induced by the current CIH protocol as had been demonstrated in previous works (Gozal et al., 2001; Xie et al., 2010), we had not conducted parallel measurement of memory and learning capability of the animals in the present study.

## Conclusion

In conclusion, for the first time, our study reveals that hippocampal neurons respond to chronic intermittent hypoxia in a complex biphasic and bidirectional manner finally leading to suppression of firing activities. Notably, these changes correlate better with impaired functions of interneurons than those of pyramidal neurons.

## Data Availability Statement

The original contributions presented in the study are included in the article/Supplementary Material, further inquiries can be directed to the corresponding author/s.

## Ethics Statement

The animal study was reviewed and approved by the Animal Experimentation and Ethics Committee of the Chinese University of Hong Kong.

## Author Contributions

YK and W-HY: conceptualization, resources, and supervision. LX, QL, and W-HY: methodology. LX: investigation and writing-original draft. LX and QL: formal analysis. LX, QL, YK, and W-HY: writing-review and editing. W-HY: funding acquisition. All authors contributed to the article and approved the submitted version.

## Funding

This work was supported by a Hong Kong RGC-CRF grant C6004-17G and the Gerald Choa Neuroscience Centre, the Chinese University of Hong Kong.

## Conflict of Interest

The authors declare that the research was conducted in the absence of any commercial or financial relationships that could be construed as a potential conflict of interest.

## Publisher's Note

All claims expressed in this article are solely those of the authors and do not necessarily represent those of their affiliated organizations, or those of the publisher, the editors and the reviewers. Any product that may be evaluated in this article, or claim that may be made by its manufacturer, is not guaranteed or endorsed by the publisher.

## Supplementary Material

Supplementary Figure 1 Single-unit spike sorting and electrophysiological identification of pyramidal neurons (PNs) and INs (INs) in CA1 region of freely behaving rats. **(A)** Verification of electrode tips by post-mortem histological staining. Scale bar: 1 mm (left); 0.1 mm (right). **(B)** The number of PNs and INs included and excluded for analysis in the control group and intermittent hypoxia group.

Supplementary Figure 2 Spike sorting and assessment of long-term stability of single-unit recordings by single microwire array over 14 days IH treatment. Example of spike sorting from single microwire array in 14 days, showing the superimposed spike waveforms of PNs and INs **(left panel)** and the inter-spike-interval histogram (ISIH, **right panel**), and the corresponding identified clusters in the PCs space (far **right panel**). Clear isolation of units from a given recording channel is indicated by high, F statistic of MANOVA (F), J3 and low Davis-Bouldin (DB) index. Scale bar : 200 μS; 58 μV.

Supplementary Figure 3 The pyramidal neurons [PNs, **(A)**] and INs [INs, **(B)**] discharge was compared between control and CIH rats. Significant decreases in neuronal firing were found toward the second week of CIH.

Supplementary Figure 4 A typical example showing that there were cyclic changes in firing oscillating at the same frequency of the hypoxia paradigm.

Supplementary Figure 5 Identification of pyramidal neurons (PNs) and interneurons (INs) in Golgi staining for quantitative analysis of spine morphogenesis. In the CA1 region of the hippocampus, PNs and INs were identified by their somatic location and somatodendritic morphologies. Scale bar: 0.4 mm.
