# Supplementary figures and images for: Chronic Intermittent Hypoxia-Induced Aberrant Neural Activities in the Hippocampus of Male Rats Revealed by Long-Term in vivo Recording
Source: Front Cell Neurosci. 2022 Jan 21;15:784045. doi: 10.3389/fncel.2021.784045 (PMC8813782; doi:10.3389/fncel.2021.784045)

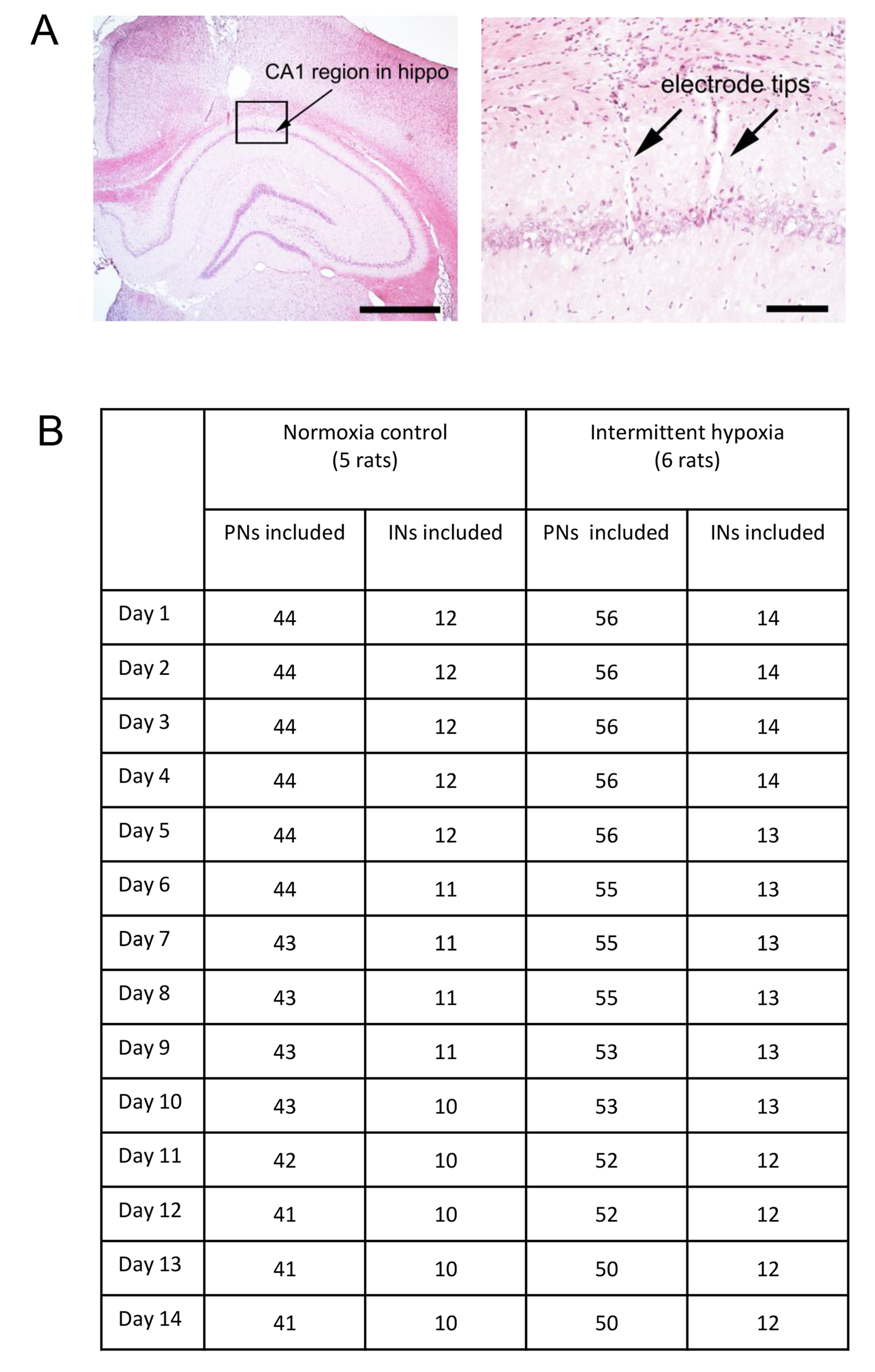

Supplement: Supplementary Figure 1 — Single-unit spike sorting and electrophysiological identification of pyramidal neurons (PNs) and INs (INs) in CA1 region of freely behaving rats. (A) Verification of electrode tips by post-mortem histological staining. Scale bar: 1 mm (left); 0.1 mm (right). (B) The number of PNs and INs included and excluded for analysis in the control group and intermittent hypoxia group. [file Image_1.JPEG]

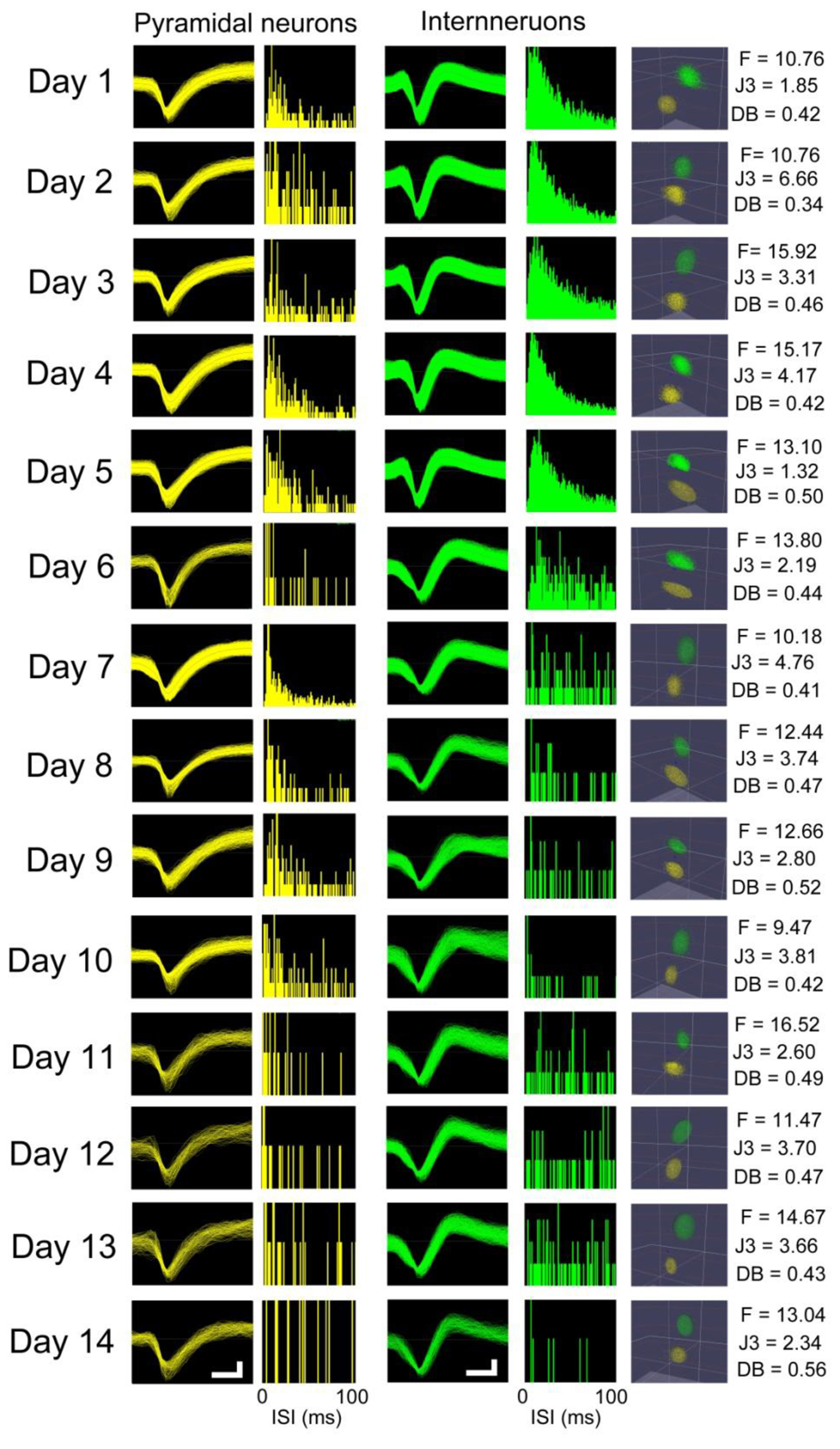

Supplement: Supplementary Figure 2 — Spike sorting and assessment of long-term stability of single-unit recordings by single microwire array over 14 days IH treatment. Example of spike sorting from single microwire array in 14 days, showing the superimposed spike waveforms of PNs and INs (left panel) and the inter-spike-interval histogram (ISIH, right panel), and the corresponding identified clusters in the PCs space (far right panel). Clear isolation of units from a given recording channel is indicated by high, F statistic of MANOVA (F), J3 and low Davis-Bouldin (DB) index. Scale bar : 200 μS; 58 μV. [file Image_2.JPEG]

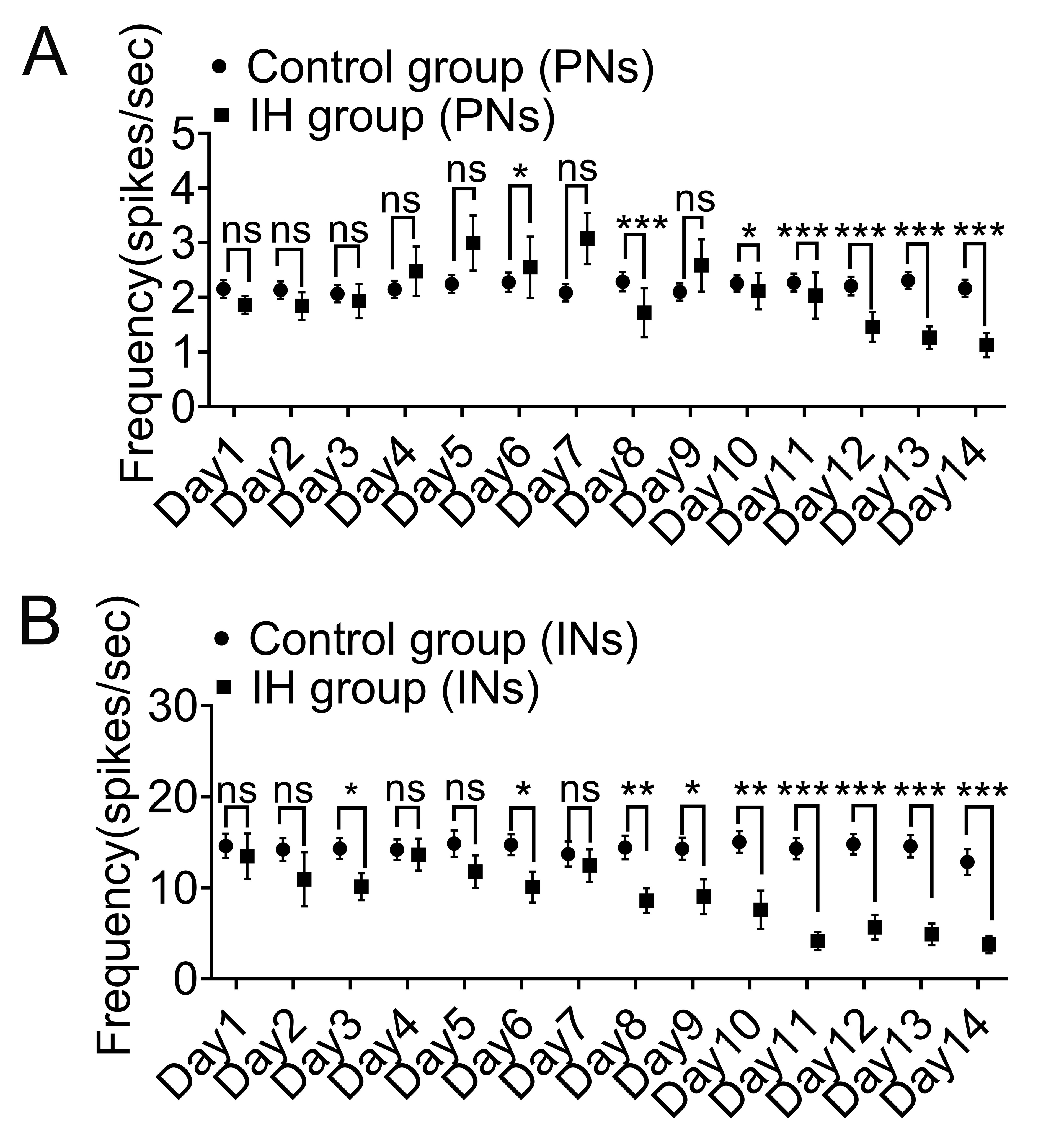

Supplement: Supplementary Figure 3 — The pyramidal neurons [PNs, (A)] and INs [INs, (B)] discharge was compared between control and CIH rats. Significant decreases in neuronal firing were found toward the second week of CIH. [file Image_3.JPEG]

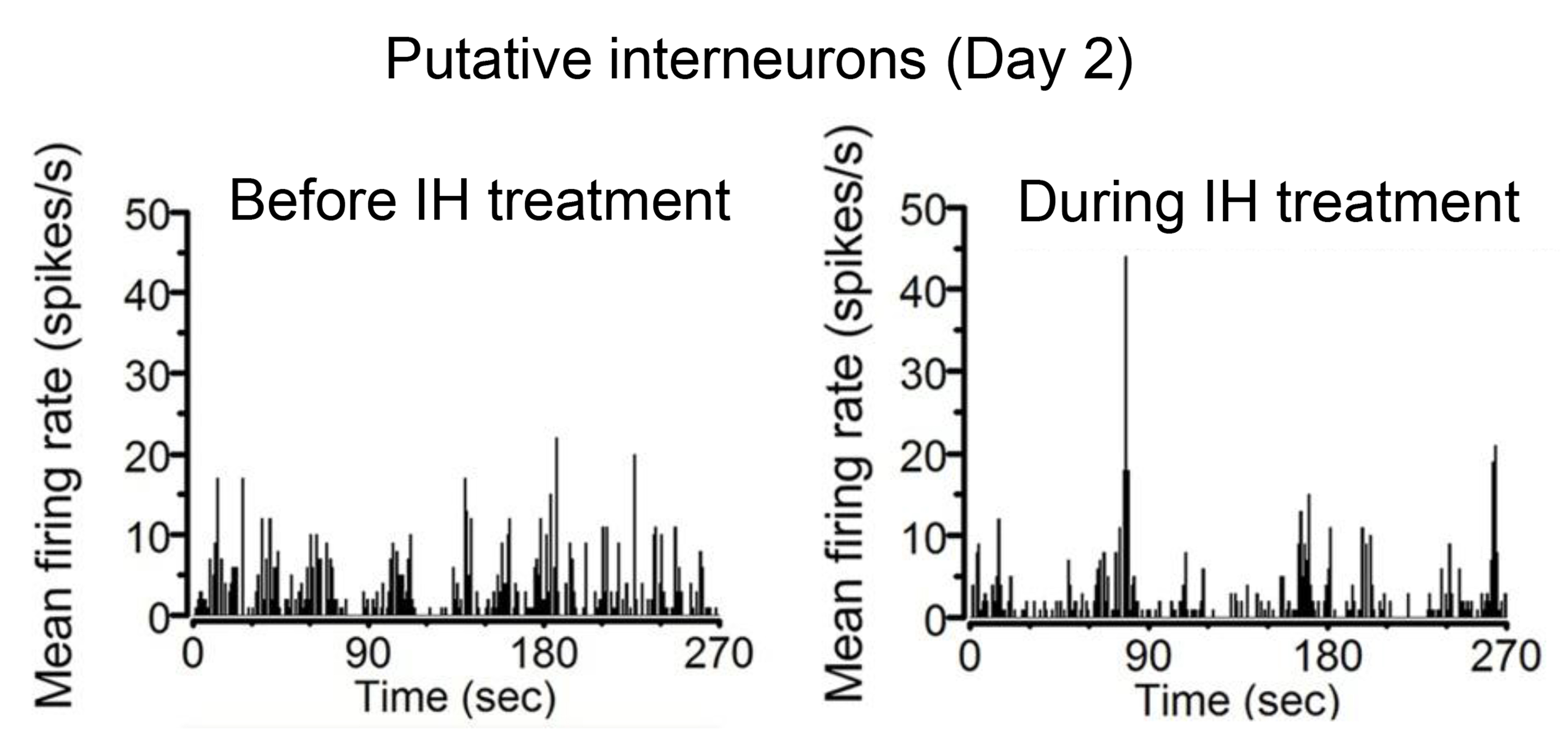

Supplement: Supplementary Figure 4 — A typical example showing that there were cyclic changes in firing oscillating at the same frequency of the hypoxia paradigm. [file Image_4.JPEG]

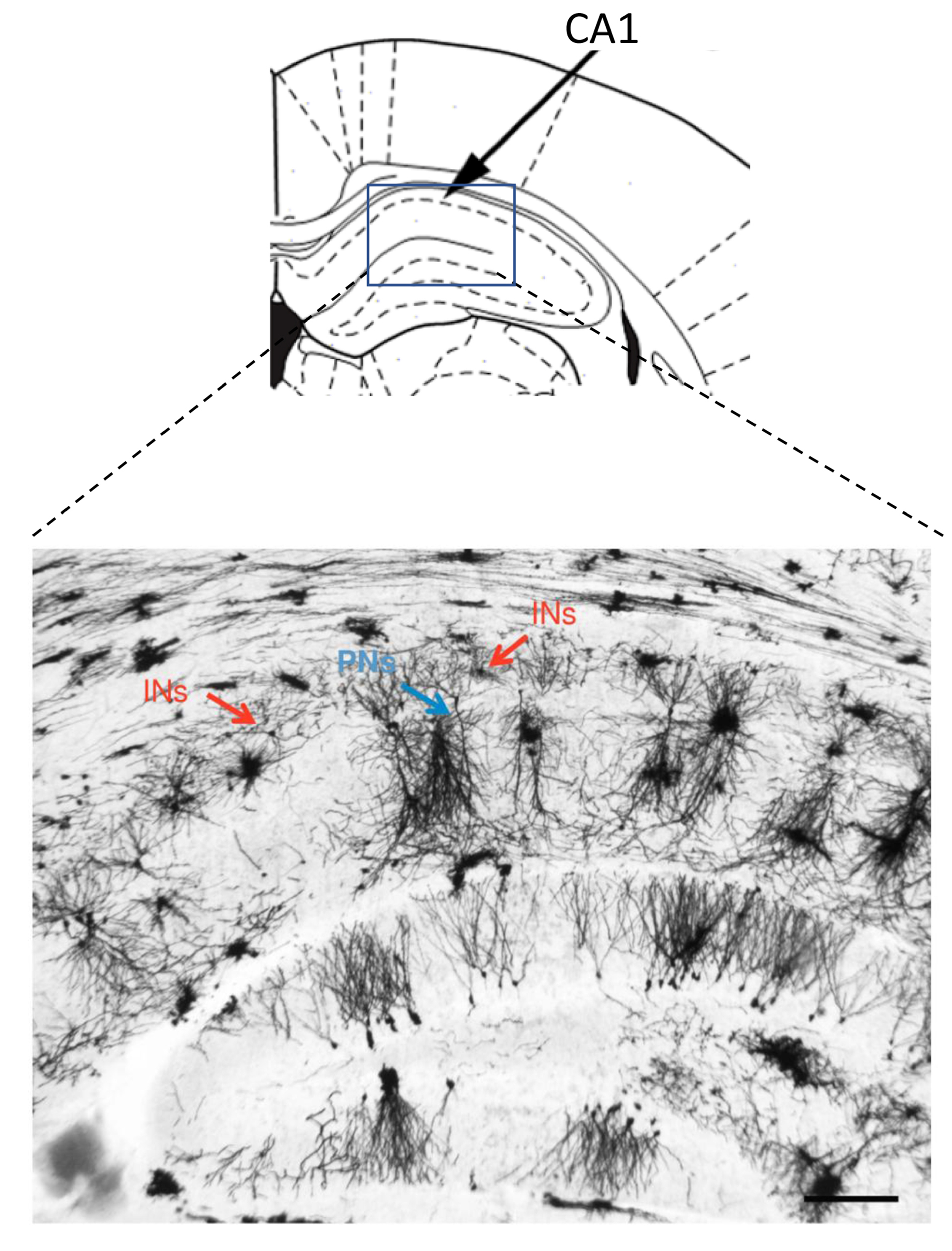

Supplement: Supplementary Figure 5 — Identification of pyramidal neurons (PNs) and interneurons (INs) in Golgi staining for quantitative analysis of spine morphogenesis. In the CA1 region of the hippocampus, PNs and INs were identified by their somatic location and somatodendritic morphologies. Scale bar: 0.4 mm. [file Image_5.JPEG]
